# Overexpression of iASPP is required for autophagy in response to oxidative stress in choriocarcinoma

**DOI:** 10.1186/s12885-019-6206-z

**Published:** 2019-10-15

**Authors:** Ka-Kui Chan, Esther Shuk-Ying Wong, Ivy Tsz-Lo Wong, Claire Ling-Yang Cheung, Oscar Gee-Wan Wong, Hextan Yuen-Sheung Ngan, Annie Nga-Yin Cheung

**Affiliations:** 1 Department of Pathology, Queen Mary Hospital, University of Hong Kong, Hong Kong SAR, China; 2 Department of Obstetrics and Gynaecology, Queen Mary Hospital, University of Hong Kong, Hong Kong SAR, China; 3 Department of Pathology, University of Hong Kong-Shenzhen Hospital, Shenzhen, China

## Abstract

**Background:**

Gestational trophoblastic disease (GTD) is a heterogeneous group of diseases developed from trophoblasts. ASPP (Ankyrin-repeat, SH3-domain and proline-rich region containing protein) family proteins, ASPP1 and ASPP2, have been reported to be dysregulated in GTD. They modulate p53 activities and are responsible for multiple cellular processes. Nevertheless, the functional role of the ASPP family inhibitory member, iASPP, is not well characterized in GTD.

**Methods:**

To study the functional role of iASPP in GTD, trophoblastic tissues from normal placentas, hydatidiform mole (HM) and choriocarcinoma were used for immunohistochemistry, whereas siRNAs were used to manipulate iASPP expression in choriocarcinoma cell lines and study the subsequent molecular changes.

**Results:**

We demonstrated that iASPP was overexpressed in both HM and choriocarcinoma when compared to normal placenta. Progressive increase in iASPP expression from HM to choriocarcinoma suggests that iASPP may be related to the development of trophoblastic malignancy. High iASPP expression in HM was also significantly associated with a high expression of autophagy-related protein LC3. Interestingly, iASPP silencing retarded the growth of choriocarcinoma through senescence instead of induction of apoptosis. LC3 expression decreased once iASPP was knocked down, suggesting a downregulation on autophagy. This may be due to iASPP downregulation rendered decrease in Atg5 expression and concomitantly hindered autophagy in choriocarcinoma cells. Autophagy inhibition per se had no effect on the growth of choriocarcinoma cells but increased the susceptibility of choriocarcinoma cells to oxidative stress, implying a protective role of iASPP against oxidative stress through autophagy in choriocarcinoma.

**Conclusions:**

iASPP regulates growth and the cellular responses towards oxidative stress in choriocarcinoma cells. Its overexpression is advantageous to the pathogenesis of GTD. (266 words).

## Background

Gestational trophoblastic disease (GTD) comprises a heterogeneous group of diseases arisen from the placental trophoblasts [1]. Hydatidiform mole (HM) is the most common form of GTD which may progress to persistent trophoblastic disease or even choriocarcinoma, a frankly malignant neoplasm and chemotherapy may be needed [2]. HM can be subclassified into partial and complete HM depending on the genetic and histopathological features. The molecular mechanism contributing to the malignant progression remains unclear. ASPP family is a group of evolutionary conserved serine-threonine kinases with three members, ASPP1, ASPP2 and iASPP, identified so far [3]. All these proteins share homology in their C-termini which are composed of ankyrin repeats, a SH3 domain and a proline-rich region. ASPP family proteins play various roles in cellular processes through affecting p53 and related proteins p63 and p73 [4]. Both ASPP1 and ASPP2 positively regulate p53-mediated activities, whereas iASPP is inhibitory on p53 functions [5]. Thus, a coordinated expression between ASPP members may be crucial for the prevention of GTD pathogenesis. We have previously demonstrated the implication of downregulation of ASPP1 and ASPP2 in GTD [5, 6]. Ectopic overexpression of these two genes triggered apoptosis in choriocarcinoma cells, whereas ASPP2 was also involved in the control of the migration potential in choriocarcinoma cells, suggesting that ASPP1/2 played a tumor suppressive role in multiple cellular functions in GTD. On the contrary, iASPP was shown to be overexpressed in various cancers and possessed anti-apoptotic functions which rendered chemoresistance [7]. Nevertheless, the oncogenic as well as other cellular effects of iASPP have yet been clearly characterized in GTD.

Autophagy refers to a process of lysosomal degradation to maintain the cellular homeostasis [8]. It is a multi-step process which is tightly regulated by numerous molecules involved at different stages. Autophagy starts from vesicle initiation by Beclin1 and VPS34, then the vesicle elongates with the coupling of Atg5 and other Atg members. Light chain (LC)3, on the other hand, is necessary for the formation of autophagosome and thus is a good indicator for autophagic activity. Fusion of autophagosome with lysosome triggers the degradation processes. Autophagy plays contradictory roles during carcinogenesis. It was thought to be a barrier for cancer initiation in breast cancer [9] but can also promote progression and chemoresistance in cancers of breast and ovary [10, 11]. The effect of iASPP on autophagy has also been investigated recently such as in regulating keratinocyte differentiation [12] but the possible interaction between iASPP and autophagy in the context of trophoblastic disease has yet been characterized. In this study, we have shown that LC3 expression was upregulated in choriocarcinoma cells when compared to normal trophoblastic cells and exhibited a close association with iASPP expression in GTD. Knockdown of iASPP decreased LC3 expression in choriocarcinoma cells. On the other hand, the ability of trophoblasts to handle the oxidative stress in pregnancy is crucial to the well-being of placenta and fetus. Autophagy is known to be an essential process induced by oxidative stress [13]. Herein, we have demonstrated that iASPP level is important for choriocarcinoma cell survival under hydrogen peroxide treatment, indicating that a functional role of iASPP on autophagy may help to deal with the oxidative stress in placenta.

## Methods

### Clinical samples and cell lines

A total of 91 normal trophoblastic tissues and GTD specimens including 10 first trimester placentas, 11 term placentas, 63 HM and 7 choriocarcinoma were used in this cohort. The patients’ age and the gestational age of HM cases ranged from 17 to 51 years and 5 to 37 weeks, respectively (gestational age of 36 cases cannot be ascertained). Follow up results were available in 39 HM with 26 cases regressed and 13 cases developed persistent trophoblastic disease requiring chemotherapy. They were retrieved from the archives of Department of Pathology, Queen Mary Hospital, Hong Kong and their corresponding clinical follow-up data were obtained. Ethical approval has been obtained from Institutional Review Board, University of Hong Kong/Hospital Authority Hong Kong West Cluster (UW 13–264) waiving need for consent.

For in vitro studies, choriocarcinoma cell lines, BeWo [American Type Culture Collection (ATCC), Manassas, VA] which was cultured in Ham’s 12 K (Kaighn’s) medium (ThermoFisher Scientific, Waltham, MA), JEG-3 and JAR cells (ATCC), were cultured in minimum essential Eagle’s medium (Caisson Labs, Smithfield, UT). HTR8/SVneo, a transformed first trimester trophoblast cell line (kindly provided by Prof. Peeyush K. Lala) [14], was cultured in RPMI 1640 medium (ThermoFisher Scientific). All media were supplemented with 10% fetal bovine serum, 100 U/ml penicillin, and 100 μg/ml streptomycin (ThermoFisher Scientific). All cell lines were cultured in a humidified incubator at 37 °C supplemented with 5% CO2.

### Transfection

For siRNA transfection, siRNA negative control and siiaspp (clone ID: s21296, s195072 and 4,390,846, ThermoFisher Scientific) at the concentration of 20 nM were used to transfect cells with siLentFect Lipid Reagent (Bio-Rad, Hercules, CA) for 24 h. The cells were then replenished with fresh, complete medium and incubated for further 48 h. For transfection of EGFP-C2-LC3 plasmid, a gift from Dr. James Murray (Trinity College Dublin, Dublin), Lipofectamine® 2000 (ThermoFisher Scientific) was used instead. The GFP signal was captured by fluorescence microscopy.

### Immunohistochemistry

Paraffin sections of 5 μm thick were cut and deparaffinized. Antigen retrieval was done by heating in Tris buffer (pH 8.0) for 10 min using a pressure cooker. Mouse monoclonal anti- iASPP antibody (Clone LXO49.3; Sigma-Aldrich, St Louis, MO) and rabbit polyclonal anti-LC3 antibody (Proteintech, Rosemont, IL) were both applied in 1:100 dilutions accordingly. The sections were incubated with antibodies at 4 °C overnight. REAL™ EnVision™ Detection System (Dako, Cambridge, UK) and DAB (3,30-diaminobenzidine tetrahydrochloride) was used to develop the signal followed by counter-staining with hematoxylin. Ovarian cancer samples with known iASPP expression status and reagent blank without primary antibody were used as positive and negative controls, respectively. Each immunostained slide per case was scanned at 20X magnification by Aperio CS2 system (Leica, Nussloch, Germany) and 4–6 regions per section were annotated for scoring with the system software ImageScope using positive pixel count v9 algorithm and generated scores as continuous values.

### Western blot

Total protein lysate was extracted with RIPA lysis buffer [50 mM Tris–HCl (pH 8.0), 150 mM NaCl, 1% (v/v) NP-40, 0.5% (w/v) deoxycholate, and 0.1% (w/v) sodium dodecyl sulfate (SDS)], supplemented with 2 mM phenylmethylsulfonyl fluoride, 1 mM sodium orthovanadate and 0.1 μM sodium okadate. Twenty μg of each sample was added and resolved by sodium dodecyl sulfate–polyacrylamide gel electrophoresis. Proteins were then transferred to polyvinylidene difluoride membrane. The membrane was blocked with 5% non-fat milk for 1 h and probed with corresponding primary antibodies at 4 °C overnight. The signal was developed with WesternBrightTM ECL (Advansta Inc., Menlo Park, CA). Rabbit polyclonal anti-LC3, p21^WAF1/Cip1^ and Atg5 antibodies were purchased from Cell Signaling Technology (Danvers, MA), while mouse monoclonal anti-iASPP and β-actin antibodies were purchased from Sigma-Aldrich. Anti-α-tubulin mouse monoclonal antibody was purchased from Santa Cruz Biotechnology (Dallas, TX).

### Quantitative reverse transcription PCR (qRT-PCR)

TRIzol reagent (ThermoFisher Scientific) was used to extract the RNA according to the manufacturer’s instruction. One μg RNA was used to synthesize cDNA with OligoDT by SuperScript™ III system (Invitrogen, Carlsbad, CA). cDNA was mixed with 2 × HotStart SYBR Green qPCR Master Mix (ExCell Bio) and 0.5 μM forward and reverse primers. The PCR reaction was 15 s at 95 °C and 45 s at 60 °C for 40 cycles in a 7900HT Fast Real-Time PCR System (Applied Biosystems). The ∆∆Ct method was used to determine the relative mRNA expression. The sequences of the primers are: p21 forward 5′ GCAGACCAGCATGACAGATTTC 3′, reverse 5’GGATTAGGGCTTCCTCTTGGA; GAPDH forward 5′ CGACAGTCAGCCGCATCTT 3′, reverse 5′ CCCCATGGTGTCTGAGCG 3′.

### MTT, clonogenic, trypan blue exclusion and BrdU incorporation assays

Choriocarcinoma cell lines were seeded in 96-well plate at a density of 6000 cells/well. Chloroquine (Sigma-Aldrich) was reconstituted in sterile water and diluted to corresponding concentrations with medium. After treatments as indicated, 10 μl MTT solution at a concentration of 5 mg/ml was added to 100 μl medium per well and incubated for 2 h at 37 °C. The formazan formed was dissolved with 100 μl DMSO and the absorbance at 570 nm was determined using Microplate Reader Infinite® 200 (Tecan, Männedorf, Switzerland). For 2D clonogenic assay, 800 cells were seeded per well in 6-well plate after transfection and were allowed to grow for 14 days. Cells were then fixed and stained in Giemsa solution (Merck, Darmstadt, Germany) containing 50% methanol for 30 min. After washing with tap water several times, colonies of at least 50 cells were counted [15]. Trypan blue exclusion assay was performed by staining cells with 0.4% trypan blue and counting cells with hemocytometer. BrdU incorporation was carried out using BrdU cell Proliferation assay kit (Cell Signaling Technology).

### Senescence detection

After cells recovered from transfection with siRNAs for 72 h, the presence of β-glalactosidase was detected by using Senescence (SA) β-Galactosidase staining kit (Cell Signaling Technology) as the manual instructed. The cell images (200X) were captured by an Inverted Microscope (Nikon Eclipse TS100). The percentage of SA-β-Galactosidase positive cells was assessed.

### Flow cytometry and TUNEL assay for apoptosis detection

Propidium iodide staining was performed. Cell pellets were collected and washed with phosphate-buffered saline (PBS), fixed with ice-cold 70% ethanol overnight, and re-suspended in PBS containing 200 μg/ml RNaseA (Thermo Fisher Scientific) and 20 μg/ml propidium iodide (Sigma-Aldrich). The samples were examined using a FACS Calibur flow cytometer (BD Bioscience, San Jose, CA). Aliquots of cells in different phases of the cell cycle were analysed with FlowJo v10. TUNEL assay was performed using In Situ Cell Death Detection Kit, Fluorescein (Sigma-Aldrich). Cells were collected and stained according to the manufacturer instruction.

### Statistical analysis

Immunohistochemical scores for the normal and GTD groups were compared with Mann-Whitney test, using SPSS version 24.0 for Windows (SPSS Inc., Chicago, IL, USA). The data generated in MTT, clonogenic and BrdU incorporation assays was examined by Student’s t-test. Spearman’s test was carried out for correlation analysis between two variables, iASPP and LC3 scores in immunohistochemical studies. Three independent experiments were performed unless specified. All data are expressed as mean ± standard error of mean (S.E.M.). A *P*-value less than 0.05 was considered statistically significant.

## Results

### Overexpression of iASPP in GTD

**Fig. 1:**
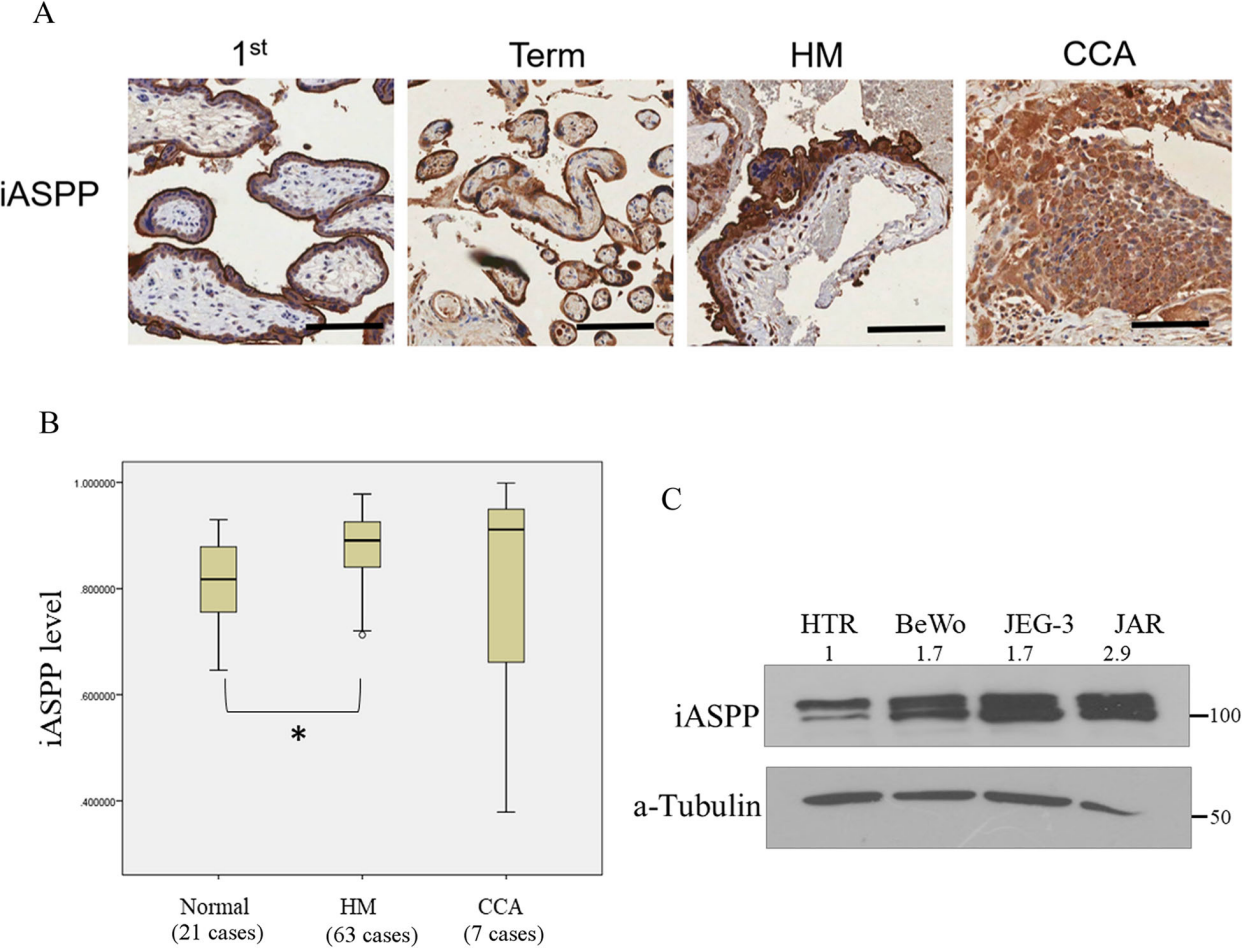
Overexpression of iASPP in GTD. **a** Photomicrographs showing higher iASPP expression level in hydatidiform moles (HM) than 1st trimester and term placenta as assessed by immunohistochemistry. Scale bar, 100 μm. **b** Statistically, higher iASPP level was demonstrated in HM than normal placenta (1st trimester and term, *P = 0.017). **c** Choriocarcinoma cell lines (BeWo, JEG-3 and JAR) showed higher iASPP expression than normal trophoblast cell line, HTR8/SV neo (HTR). Total forms of iASPP (both phospho and unphospho- forms) were detected and their relative intensities normalized with actin were measured by ImageJ and depicted as numbers on the top

We have previously demonstrated that HM or choriocarcinoma had lower ASPP1 and ASPP2 expression than normal placentas [5, 6]. Here, we evaluated the endogenous iASPP level in GTD samples. The iASPP protein expression was predominantly found at the cytoplasm. In contrast to ASPP1 and ASPP2, HM expressed significantly higher (*P* < 0.001) iASPP than first trimester and term placentas (Fig. 1a&b). There was, however, no statistically significant difference in iASPP immunoscores between HM that spontaneously regressed (*n* = 26) and those developed persistent trophoblastic disease requiring chemotherapy (*n* = 13) (*P* = 0.231). The mean iASPP expression of those progressive cases was 0.91 which was slightly higher than that of regressed cases (0.89). Choriocarcinoma had the highest iASPP score among all sample types but statistical significance cannot be reached when compared to normal placenta or HM (Fig. 1b). This may be due to the diverse status of chemotherapy among those choriocarcinoma cases. Three choriocarcinoma cell lines, BeWo, JEG-3 and JAR, were also used to compare iASPP expression to that in a normal trophoblast cell line, HTR8/SVneo. Consistently, a higher iASPP expression was found in all choriocarcinoma cell lines compared with HTR8/SVneo cells (Fig. 1c).

### Functional importance of iASPP on the growth of choriocarcinoma cells

**Fig. 2:**
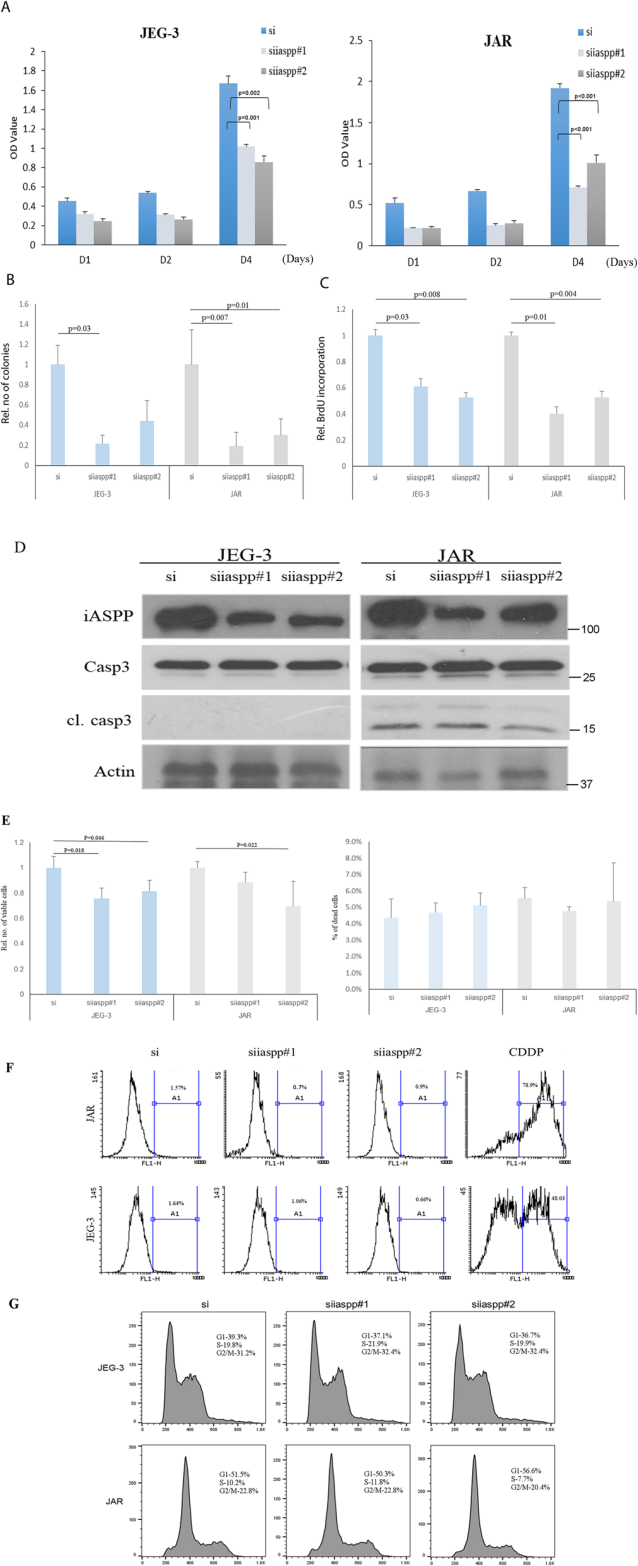
Knockdown of iASPP affected the growth of choriocarcinoma cells. **a** Choriocarcinoma cells with iASPP knockdown by using two siRNA (siiaspp#1 & siiaspp#2) grew slower than those transfected with scramble control (si) as indicated in MTT assay. (For JEG-3, si vs siiaspp#1, *P* = 0.001; si vs siiaspp#2, *P* = 0.002. For JAR, both si vs siiaspp, *P* < 0.001). **b** Both JEG-3 and JAR cells with iASPP silencing formed less colonies than the scramble control. (For JEG-3, si vs siiaspp#1, *P* = 0.03; si vs siiaspp#2, *P* = 0.19]; For JAR, si vs siiaspp#1, *P* = 0.007, si vs siiaspp#2, *P* = 0.01). **c** The incorporation of BrdU was less in choriocarcinoma cells with iASPP knockdown than the scramble control. (For JEG-3, si vs siiaspp#1, *P* = 0.03; si vs siiaspp#2, *P* = 0.008; For JAR, si vs siiaspp#1, *P* = 0.01; si vs siiaspp#2, *P* = 0.004 (**d**) No apparent increase in cleaved caspase 3 (cl. casp3) was seen after iASPP was downregulated. **e** Trypan blue exclusion assay was used to assess the number of viable cells (left panel) and dead cells (right panel) under different transfection conditions. **f** TUNEL assay was used to measure the presence of DNA breaks. Cisplatin (CDDP, 10 μM for 24 h) treated cells were used as positive controls. **g** Histograms showing different cell cycle phases of choriocarcinoma cells with or without iASPP knockdown

**Fig. 3:**
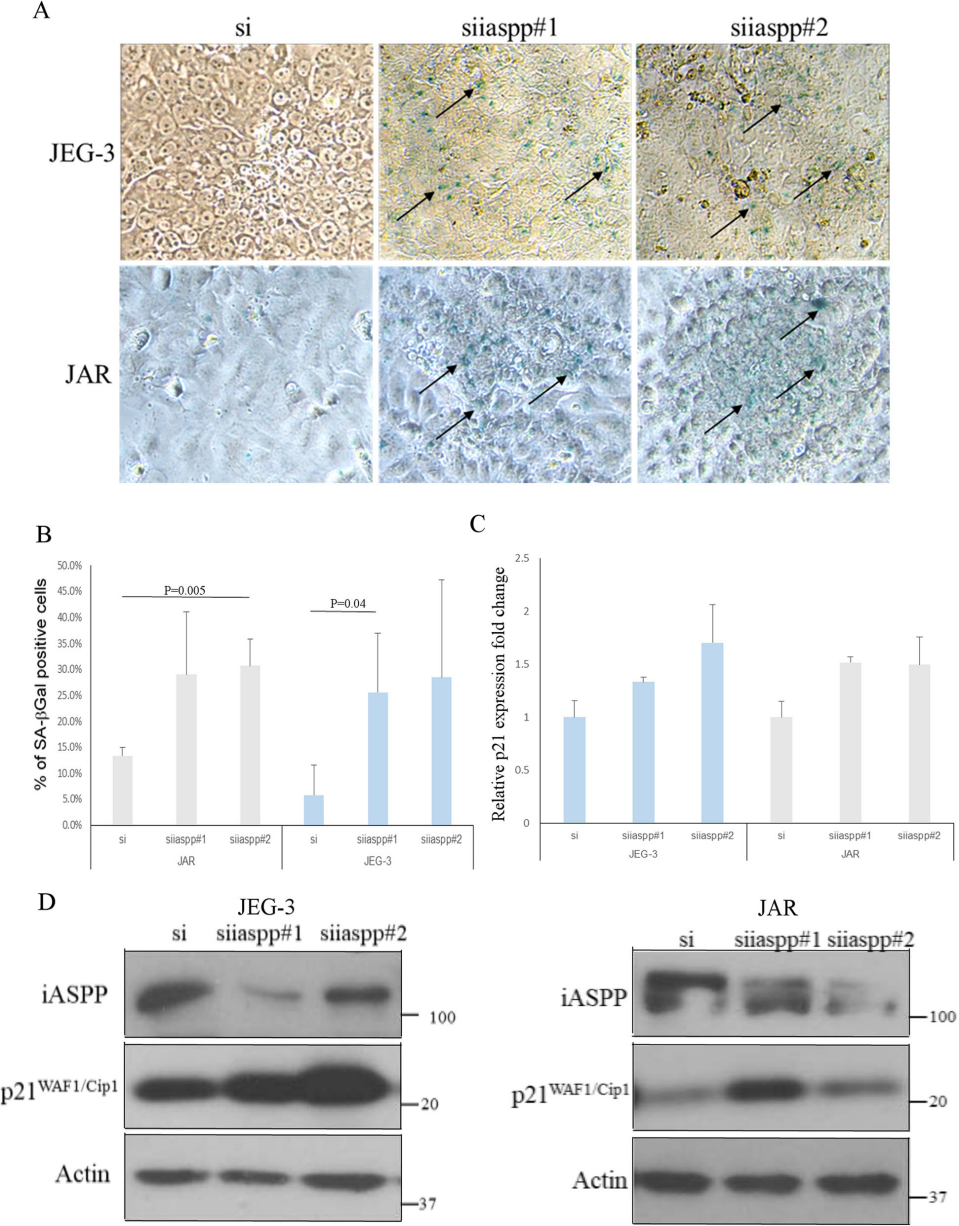
Knockdown of iASPP induced senescence in choriocarcinoma cells. **a** The presence of senescent cells was detected by SA-β-Galactosidase staining (blue color, indicated with arrows) in both JEG-3 and JAR cells with iASPP knockdown (200X magnification). **b** The percentage of SA-β-Galactosidase positive cells was measured and compared. Statistically significant increase in senescence was found in JEG-3, si vs siiaspp#1 (*P* = 0.04), and JAR, si vs siiaspp#2 (*P* = 0.005), respectively. **c** Increase in p21^WAF1/Cip1^ mRNA levels in JEG-3 and JAR cells with iASPP knockdown as measured by qRT-PCR. **d** At protein level, knockdown of iASPP also induced the expression of p21^WAF1/Cip1^ in choriocarcinoma cells

Two independent siRNAs (siiaspp#1& siiaspp#2) were used to knock down the iASPP expression in choriocarcinoma cell lines JEG-3 and JAR. Silencing iASPP in JEG-3 and JAR cells decreased their growth as less viable cells and colonies were illustrated in MTT and clonogenic assays, respectively (Fig. 2a&b). The effects were likely exerted by inhibition on cell proliferation rather than apoptosis induction. Less BrdU incorporation was observed in choriocarcinoma cells after iASPP knockdown, suggesting a decrease in DNA synthesis upon iASPP downregulation (Fig. 2c). No increase in cleaved caspase 3 protein expression could be detected after iASPP knockdown as well (Fig. 2d). Decrease in cell viability after iASPP knockdown was corroborated by trypan blue exclusion assay (Fig. 2e). On the contrary, no remarkable increase in DNA breaks and fragmentation were observed in iASPP knockdown cells as detected by TUNEL and PI staining assays, respectively (Fig. 2f &g). More importantly, senescence was induced after iASPP silencing. More cells with iASPP downregulation were stained with SA-β-Gal than scramble control (Fig. 3a&b). Higher mRNA and protein expression of p21^WAF1/Cip1^, a CDK inhibitor which is p53 dependent, was expressed in cells with iASPP knockdown, corroborating the induction of senescence (Fig. 3c&d). All these evidence suggest that iASPP affects the growth of choriocarcinoma cells.

### Functional relationship between iASPP and autophagy in GTD

**Fig. 4:**
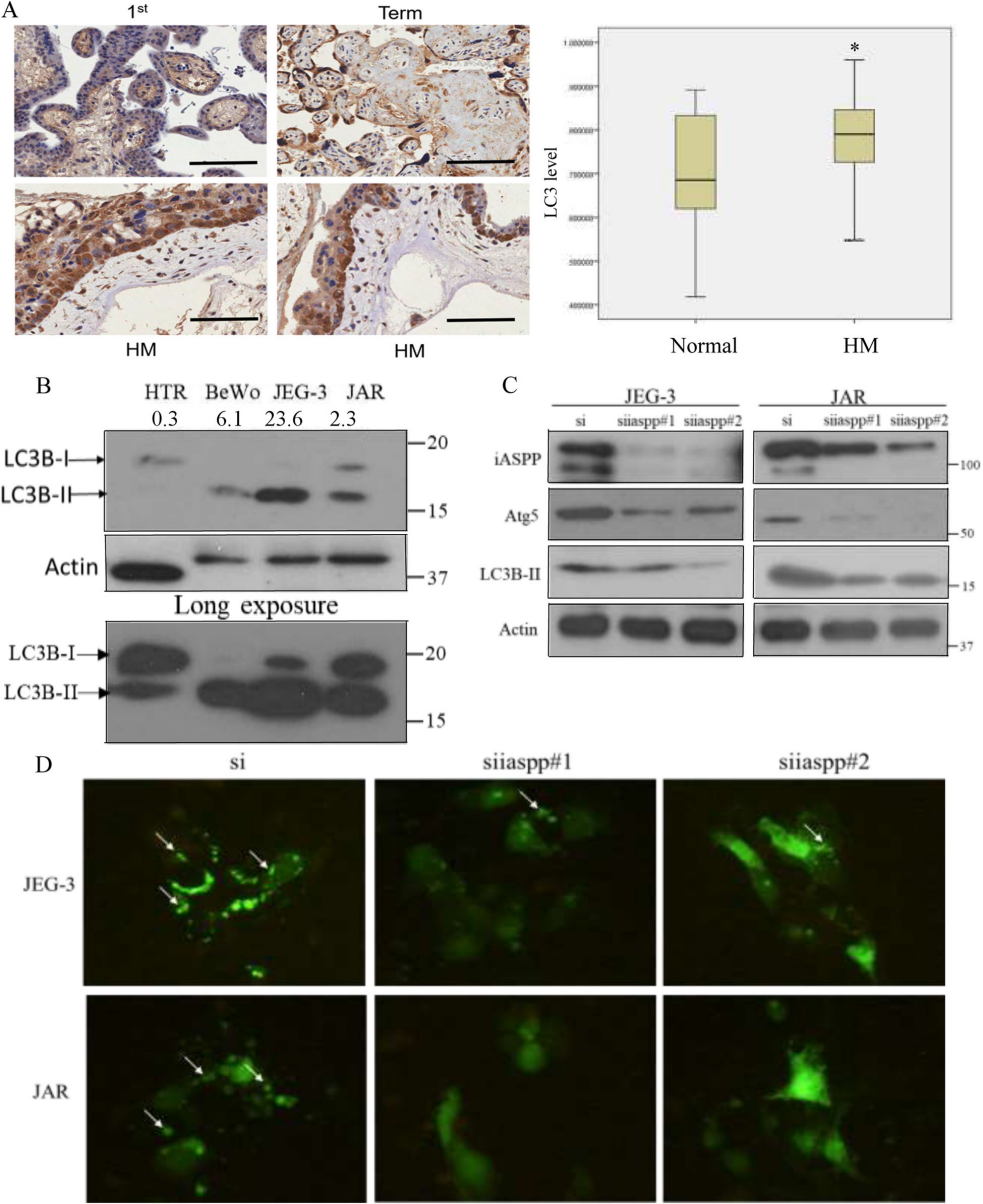
Effect of iASPP on autophagy. **a** Higher expression of LC3 was demonstrated in HM than normal placentas by immunohistochemistry (normal vs HM *; *P* = 0.043). Scale bar, 100 μm. **b** Choriocarcinoma cell lines, BeWo (2), JEG-3 (3) and JAR (4), expressed more LC3-II than normal trophoblast cell line HTR8/SVneo (1). The LC3 bands were quantified by using ImageJ. The LC3-II to LC3-I ratio on each cell line was analyzed and listed. **c** Knockdown of iASPP in choriocarcinoma cells decreased Atg5 and LC3-II expressions. **d** More GFP-LC3 puncta (arrows) were observed in choriocarcinoma cells treated with scramble control than those cells with iASPP knockdown. All the cells were treated with Bafilomycin A1 (20 nM) for 6 h before captured under microscope (200X)

We have also evaluated the effect of iASPP on autophagy. Endogenous level of LC3 is closely associated with the autophagic activity. In general, HM samples expressed significantly higher (*P* = 0.043) LC3 level than normal placenta (Fig. 4a). Processing of LC3 during autophagy is a good readout for autophagic activity [16]. LC3 is firstly cleaved into cytosolic LC3-I which is then lipidated to form LC3-II on the membrane of autophagosome during an autophagic flux. Thus, an increase in LC3-II to LC3-I expression ratio indicates a more active autophagy. Consistent with the immunohistochemistry results, the overall expression of LC3 (I & II) was higher in all choriocarcinoma cells BeWo, JEG-3 and JAR while they also attained higher LC3-II to LC3-I ratio than that in HTR8/SVneo cells (Fig. 4b). All together suggests that autophagy may be more active in choriocarcinoma cells. Moreover, iASPP expression was significantly associated with LC3 expression in HM tissues, as assessed immunohistochemically (Pearson correlation = 0.419, *P* = 0.001).

Choriocarcinoma cells with iASPP downregulation presented less LC3-II expression than scramble control with the absence of LC3-I in all samples (Fig. 4c). Autophagosome formation can be illustrated by the presence of LC3 puncta and act as an indication of active autophagy. Bafilomycin A1, a lysosomal inhibitor, was added and resulted in the formation of GFP-LC3 puncta. Less puncta was observed in choriocarcinoma cells with iASPP silencing by fluorescence microscopy (Fig. 4d). All these evidence suggest an obstruction on autophagy upon iASPP downregulation. Such regulation on autophagy may be mediated by Atg5 which is responsible for autophagosomal membrane formation, and its downregulation has been shown to affect the autophagy [17]. Indeed, we here observed that iASPP downregulation reduced Atg5 expression in both JEG-3 and JAR cells (Fig. 4c) that may lead to suppression on autophagic function.

### Silencing of iASPP or autophagy inhibition sensitized choriocarcinoma cell towards oxidative stress

**Fig. 5:**
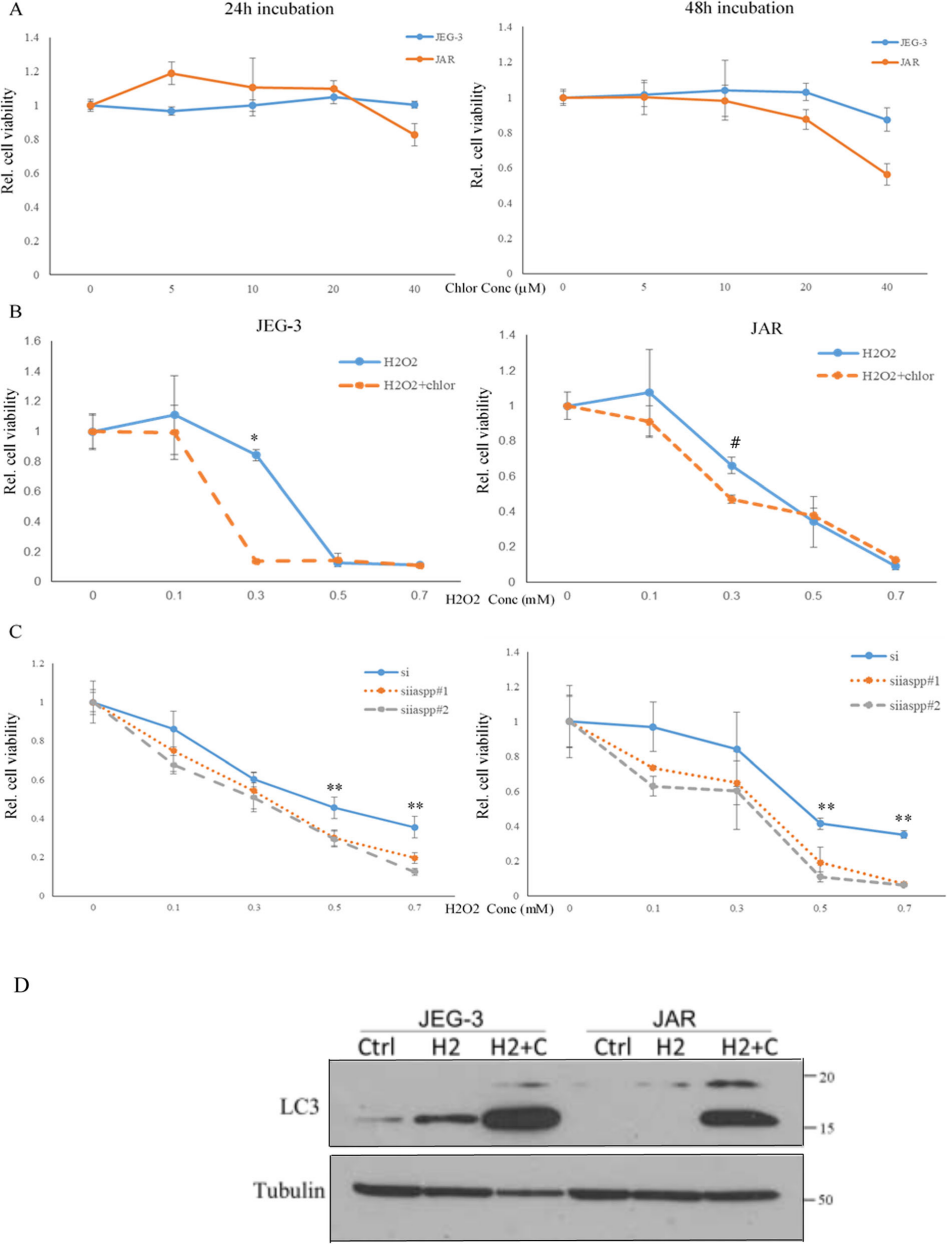
Autophagy inhibition sensitized choriocarcinoma cells in response to hydrogen peroxide. **a** Chloroquine inhibition on autophagy per se had no effect on the viability of choriocarcinoma cells as measured by MTT assay in the first 24 h. **b** Addition of chloroquine (20 μM) enhanced the toxic effect of hydrogen peroxide at low dosage after 24 h (*P < 0.001; ^#^*P* = 0.02). **c** Choriocarcinoma cells with iASPP knockdown (siiaspp#1/2) were more sensitive to hydrogen peroxide than scramble control (si). **d** Change of LC3 expression after addition of hydrogen peroxide (H2, 0.3 mM) with or without chloroquine (C, 20 μM). (** *P* < 0.05)

**Fig. 6:**
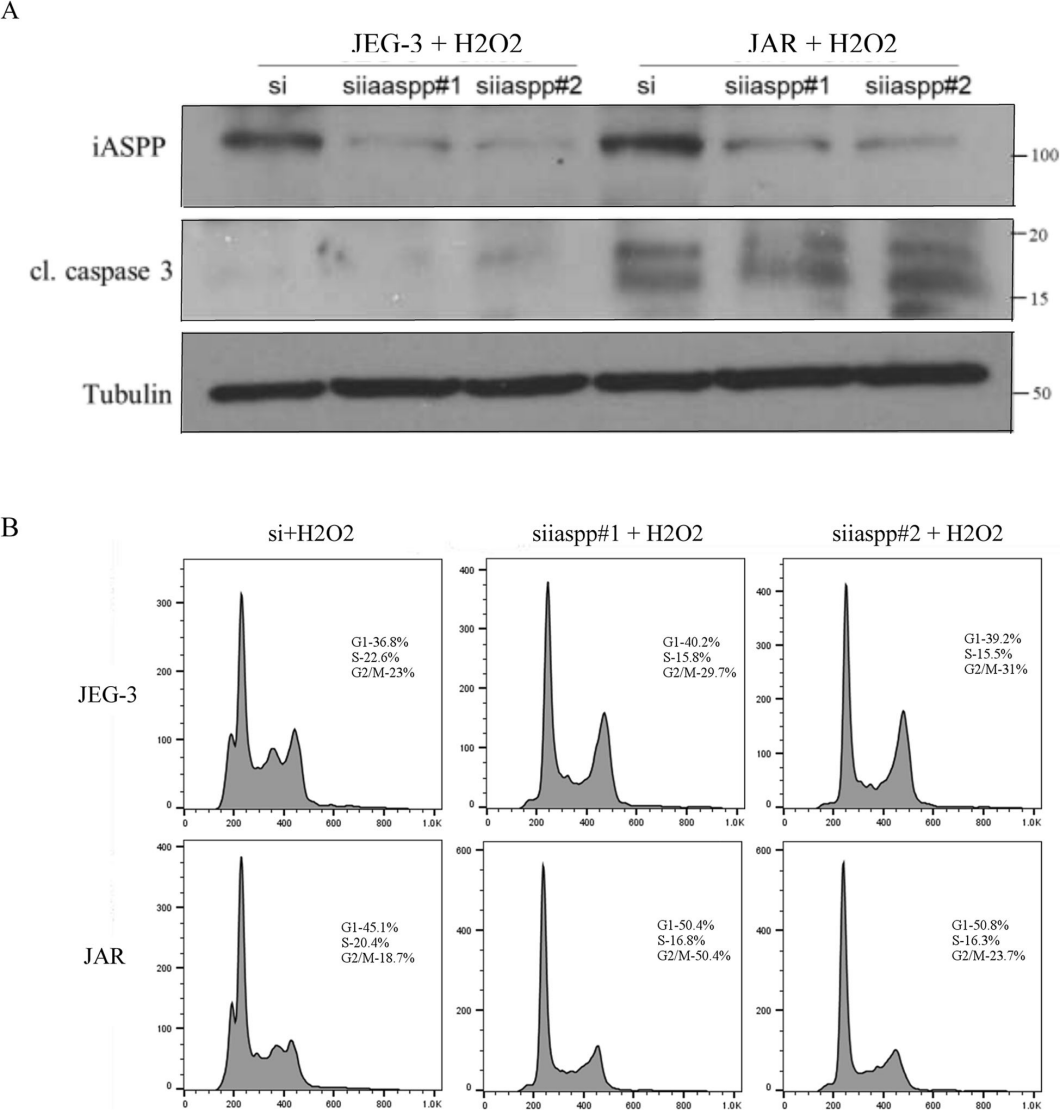
Increased proportion of cells at G_2_/M in choriocarcinoma cells with iASPP silencing when treated with hydrogen peroxide. **a** No obvious increase in cleaved caspase 3 between scramble control and iASPP knockdown cells. **b** The G_2_/M population in cells with iASPP silencing was higher than the scramble control when they were all treated with hydrogen peroxide_._ However, no statistically significant difference was achieved_._ Representative results of two independent experiments were shown

Chloroquine, a clinically used lysosomal inhibitor, was also effective in blocking autophagy. In the context of choriocarcinoma, chloroquine did not show strong impact on the cell viability during a 24 h incubation period unless a high concentration (40 μM) was used (Fig. 5a). Autophagy is usually induced under oxidative stress, the consequence of which can be protective or detrimental depending on the cell context [18]. Hydrogen peroxide is a strong oxidizing agent that can induce apoptosis [19]. By using a sub lethal dose of chloroquine (20 μM), addition of hydrogen peroxide (H_2_O_2_) along with chloroquine resulted in less viable cells when compared to treatment with H_2_O_2_ alone especially at lower dose (Fig. 5b), suggesting that autophagy inhibition sensitizes choriocarcinoma cells to oxidative stress. Chloroquine blocks autophagic flux and leads to accumulation of LC3-II. We found that H_2_O_2_ slightly increased LC3-II in JEG-3 cells, whereas it resulted in the highest LC3-II expression when chloroquine was added simultaneously (Fig. 5d). Similarly, choriocarcinoma cells with iASPP knockdown were more sensitive to H_2_O_2_ inhibition with more reduction in cell viability than the scramble control under a wider range of concentration (Fig. 5c). The decrease in viable cell was unlikely due to an induction of apoptosis as the levels of cleaved caspase 3 were comparable among different treatment groups (Fig. 6a). Instead, we found that the cell proliferation was affected as cells with silencing iASPP accumulated more in G_2_/M phase once treated with H_2_O_2_ when compared to scramble controls (Fig. 6b).

## Discussion

iASPP is a discrete member of the ASPP family with respect to its functions on p53 and p63 activation. Structurally, iASPP lacks the α–helical domain which is present in other members ASPP1/2 [4]. Functionally, iASPP is considered anti-apoptotic and oncogenic, whereas ASPP1/2 is pro-apoptotic and tumor suppressive. Thus, dysregulation on ASPP expression may be common but different among diseases [7, 20]. Our previous studies have also demonstrated the downregulation of ASPP1/2 in GTD [5, 6]. Here, we showed higher expression of iASPP in HM and choriocarcinoma when they were compared to normal placenta although there was no significant correlation between iASPP expression level and the progression or regression of HM, suggesting that iASPP may not be a good predictive marker for HM progression. Altogether, it seems that an imbalanced expression between iASPP (upregulated) and ASPP1/2 (downregulated) is important for the pathogenesis of GTD.

The primary role of iASPP in apoptosis has been well characterized in cancer cells. In recent years, more alternative functions of iASPP have emerged. Here we show that iASPP also plays a role in cellular senescence. ASPP family members have been reported to participate in senescence through mediating the activities of different p53 family members [21, 22]. In the context of choriocarcinoma, we also showed that iASPP deficiency triggered senescence through the induction of p21^WAF1/Cip1^ expression to suppress cell growth but not through its well established anti-apoptotic effect. A high iASPP level may prevent p53 to induce senescence through the transcription of p21^WAF1/Cip1^. Direct binding of p53 to the promoter region of p21^WAF1/Cip1^ and activation of its transcription has been demonstrated [23]. In a more recent study, overexpression of antiproliferative gene, TIS21, though inhibited p53-iASPP interaction, shifted p53-induced senescence to apoptosis through posttranslational modification of p53 [24], suggesting that additional mediators are involved in determination of p53-induced senescence or apoptosis. In contrast, another group showed iASPP silencing reduced p21^WAF1/Cip1^ expression in keratinocytes and promoted terminal differentiation through an iASPP-p63 feedback loop mechanism [21]. Such discrepancy indicates that depending on the cell context and mediators iASPP interacts, different cellular responses may result.

iASPP has also been illustrated to regulate autophagy in keratinocytes [12]. On the contrary to the inhibitory effect in keratinocytes, iASPP may be necessary for maintaining an active autophagy in choriocarcinoma cells via regulating the Atg5 expression. The positive correlation between iASPP and LC3 expressions in clinical samples further suggested a possible link between iASPP and autophagy in GTD. Autophagy is important for cellular homeostasis and its dysregulation has been found in various diseases [25]. Autophagy was firstly linked to tumorigenesis when monoallelic deletion of Beclin1, a modulatory gene on autophagy, was found in breast and ovarian cancers [26]. In contrast, studies have also demonstrated that autophagy inhibition enhanced cytotoxic effects of chemotherapy but promoted proliferation in certain cellular context [27, 28], suggesting that autophagy may play a role in cancer survival under stress. Autophagy provides not only the nutrients and energy but also the cellular restructuring in response to metabolic stress. Such paradox on autophagy effect is mainly because autophagy participates in processes promoting cell death and cell survival [29, 30], indicating that a tight regulation on autophagy is crucial. Based on a higher LC3-II to LC3-I expression ratio and LC3 level found in choriocarcinoma cells and HM respectively, it is likely that an upregulated autophagy may exhibit pro-survival effect for GTD. Active autophagy is proven to be necessary for the progression in other cancer types [31].

Our evidence showing autophagy promoting effect of iASPP in choriocarcinoma was different from studies on keratinocytes where iASPP was shown to be an autophagy inhibitor in keratinocytes [12]. Such discrepancy may be mainly due to the differences in the nature of cells. Choriocarcinoma cells have a high basal autophagy activity as we noticed choriocarcinoma cell lines expressing higher LC3-II level than normal trophoblastic cell. We have shown that iASPP knockdown suppressed expression of Atg5 and subsequent GFP-LC3 puncta formation. Atg5 is responsible for autophagosome formation [32]. Overexpression of Atg5 has been shown to activate autophagy, whereas knockdown of Atg5 resulted in autophagic downregulation. A recent study also reported that knockdown of iASPP downregulated autophagy in lung cancer cells through interfering the autophagosome formation [33].

Interestingly, we also demonstrated that either iASPP silencing or autophagy inhibition sensitized choriocarcinoma cells towards oxidative stress induced by hydrogen peroxide. Crosstalk between autophagy and oxidative stress signal has been reported [13]. Generation of hydrogen peroxide activates AMPK and triggers the initiation of autophagy [34] which is found to be cytoprotective for cells in response to oxidative stress [35]. Blockage of autophagy with chloroquine may prevent protecting choriocarcinoma cells from the oxidative stress induced by hydrogen peroxide. This provides a novel therapeutic approach against choriocaricoma. Silencing iASPP may also render choriocarcinoma cells more susceptible to hydrogen peroxide through regulating autophagy. Further investigation is needed to delineate the underlying mechanisms by identifying the common mediators affected by iASPP knockdown and chloroquine in future studies. However, we should not exclude other factors such as effects of cell cycle alteration. A recent study has shown that iASPP regulates the recruitment of CEP55 to the midbody and concomitantly controls cytokinesis [36]. In addition, oxidative stress can induce mitotic arrest [37], suggesting that cell cycle may be potently deregulated when hydrogen peroxide is applied to cells with iASPP deficiency. Our findings on cell cycle analysis also show an apparent increase in the proportion of cells at G_2_/M phase under hydrogen peroxide treatment in iASPP knockdown cells, despite statistical significance was not achieved. On the other hand, iASPP has recently reported as an antioxidative factor to participate in regulating the reactive oxygen species homeostasis [38], again supporting that iASPP may also play a role in regulation of oxidative stress in GTD.

## Conclusions

iASPP may be a potential therapeutic target for choriocarcinoma as iASPP silencing not only inhibits cell growth but also renders higher susceptibility to oxidative stress.

## Supplementary information

**Additional file 1.** A list of average immunoscores for patients and their individual diagnosis.

**Additional file 2.** The raw western blots for iASPP shown in the manuscript.

## Data Availability

A table listing cases for immunohistochemical evaluation is provided as Additional files 1 and 2.

## Abbreviations

ASPP: Ankyrin-repeat, SH3-domain and proline-rich region containing protein
BrdU: 5-bromo-2′-deoxyuridine
GTD: gestational trophoblastic disease
HM: hydatidiform mole
LC3: Light chain 3
MTT: 3-(4, 5-dimethylthiazolyl-2)-2, 5-diphenyltetrazolium bromide
