# Supplementary material for: Overexpression of iASPP is required for autophagy in response to oxidative stress in choriocarcinoma
Source: BMC Cancer. 2019 Oct 15;19:953. doi: 10.1186/s12885-019-6206-z (PMC6792270; doi:10.1186/s12885-019-6206-z)
Supplement: Supplementary file 1 — Additional file 1. A list of average immunoscores for patients and their individual diagnosis. [file 12885_2019_6206_MOESM1_ESM.pdf]

Case no	Diagnosis	Regress (1); Progress (2)	Metastasis	Average iAspp score	Average LC3B score
1	HM	n.a.	n.a.	0.930856	0.759062
2	HM	n.a.	n.a.	0.874093	0.529198
3	HM	n.a.	n.a.	0.831175	0.416546
4	HM	1	1	0.891034	0.765213
5	HM	1	1	0.890321	0.815747
6	HM	n.a.	n.a.	0.816655	0.547529
7	HM	n.a.	n.a.	0.736066	0.585588
8	HM	n.a.	n.a.	0.921361	0.845401
9	HM	n.a.	n.a.	0.819149	0.673977
10	HM	n.a.	n.a.	0.793870	0.597205
11	HM	n.a.	n.a.	0.712830	0.731104
12	HM	n.a.	n.a.	0.767316	0.505537
13	HM	n.a.	n.a.	0.720601	0.612745
14	HM	1	1	0.899673	0.880954
15	HM	n.a.	n.a.	0.944275	0.710757
16	HM	n.a.	n.a.	0.824266	0.818044
17	HM	n.a.	n.a.	0.848876	0.797085
18	HM	n.a.	n.a.	0.901833	0.737744
19	HM	n.a.	n.a.	0.940994	0.529192
20	HM	n.a.	n.a.	0.836106	0.732885
21	HM	1	1	0.900076	0.740864
22	HM	1	1	0.900509	0.838369
23	HM	1	1	0.920642	0.812325
24	HM	2	2	0.840429	0.854675
25	HM	2	1	0.814554	0.815823
26	HM	1	1	0.859252	0.847136
27	HM	1	1	0.832148	0.768838
28	HM	1	1	0.889190	0.932620
29	HM	n.a.	n.a.	0.977910	0.946788
30	HM	n.a.	n.a.	0.954856	0.959546
31	HM	2	1	0.906612	0.932714
32	HM	1	1	0.877684	0.771339
33	HM	1	1	0.888272	0.929007
34	HM	1	1	0.881902	0.790188
35	HM	1	1	0.872151	0.883322
36	HM	n.a.	n.a.	0.830953	0.944982
37	HM	n.a.	n.a.	0.840840	0.868133
38	HM	1	1	0.870134	0.659038
39	HM	1	1	0.729137	0.659687
40	HM	1	1	0.947475	0.873975
41	HM	1	1	0.924436	0.841637
42	HM	2	2	0.873383	0.766947
43	HM	2	1	0.973458	0.721662
44	HM	2	1	0.920924	0.756897
45	HM	1	1	0.961956	0.803279

46	HM	1	1	0.960186	0.850244
47	HM	1	1	0.943407	0.822814
48	HM	2	1	0.968043	0.837464
49	HM	2	1	0.925828	0.637234
50	HM	2	2	0.961358	0.855461
51	HM	1	1	0.868659	0.694788
52	HM	2	1	0.965135	0.898219
53	HM	1	1	0.949995	0.623895
54	HM	2	2	0.908073	0.788455
55	HM	2	1	0.861395	0.782556
56	HM	1	1	0.900272	0.833110
57	HM	1	1	0.924922	0.814877
58	HM	1	1	0.902200	0.819352
59	HM	1	1	0.930116	0.872989
60	HM	1	1	0.767185	0.788629
61	HM	n.a.	n.a.	0.856247	0.786474
62	HM	2	1	0.917523	0.827793
63	HM	n.a.	n.a.	0.866158	0.738178

n.a. not available

Normal placenta and choriocarcinoma

Case no	Diagnosis			Average iAspp scor	verage LC3B score
1	Term			0.686320	0.611711
2	Term			0.735378	0.588255
3	Term			0.817650	0.833008
4	Term			0.729566	0.892091
5	Term			0.841093	0.713212
6	Term			0.646102	0.683173
7	Term			0.744603	0.842802
8	Term			0.878177	0.885737
9	Term			0.835083	0.880291
10	Term			0.810633	0.814179
11	Term			0.853359	0.687227
12	1st			0.773019	0.674726
13	1st			0.891208	0.664781
14	1st			0.913384	0.620295
15	1st			0.782955	0.576681
16	1st			0.929519	0.863757
17	1st			0.837406	0.712281
18	1st			0.886375	0.652149
19	1st			0.791320	0.418343
20	1st			0.755834	0.526463
21	1st			0.892049	0.632831
22	CCA			0.998871	n.d.
23	CCA			0.779285	n.d.
24	CCA			0.926275	n.d.
25	CCA			0.378917	n.d.

26	CCA			0.543351	n.d.
27	CCA			0.972303	n.d.
28	CCA			0.910740	n.d.

n.d. not determined
