# Supplementary material for: Overexpression of iASPP is required for autophagy in response to oxidative stress in choriocarcinoma
Source: BMC Cancer. 2019 Oct 15;19:953. doi: 10.1186/s12885-019-6206-z (PMC6792270; doi:10.1186/s12885-019-6206-z)
Supplement: Supplementary file 2 — Additional file 2. The raw western blots for iASPP shown in the manuscript. [file 12885_2019_6206_MOESM2_ESM.pdf]

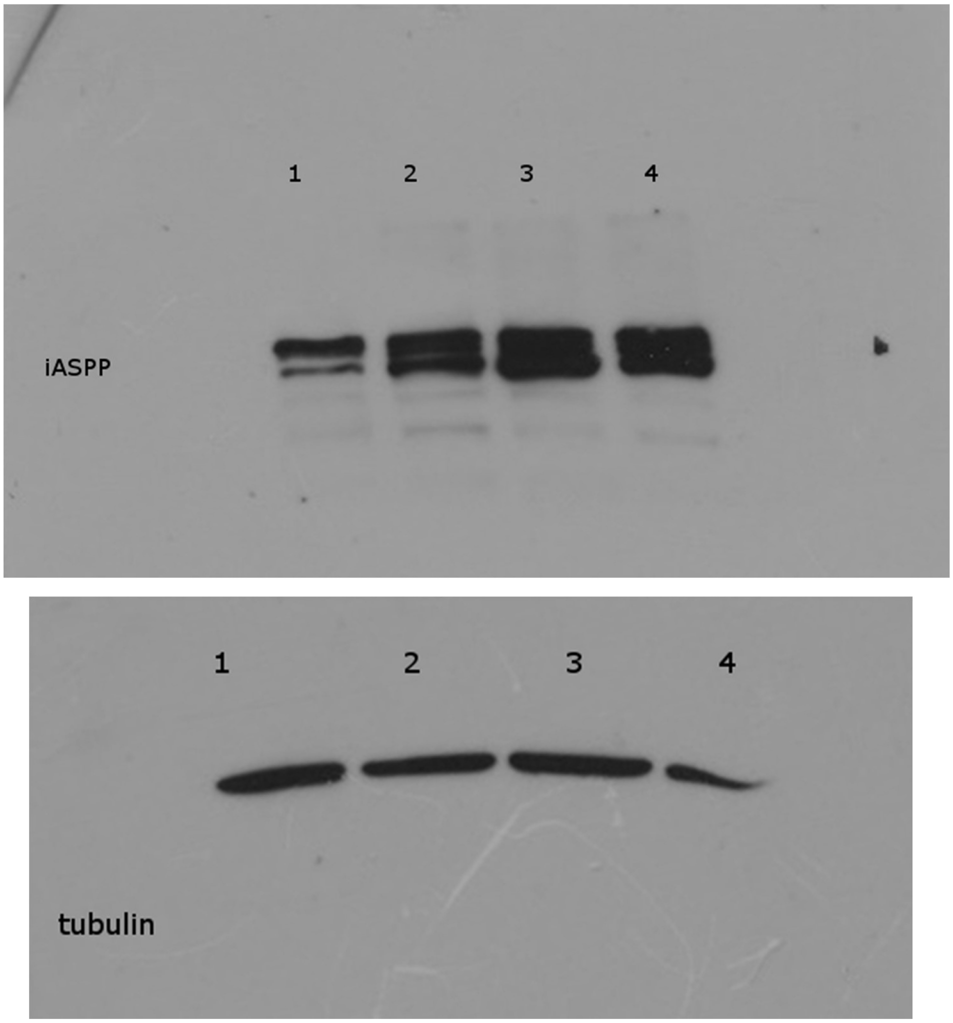

Figure 1C. Lane 1-HTR, Lane 2-BeWo, Lane 3-JEG-3, Lane 4-JAR

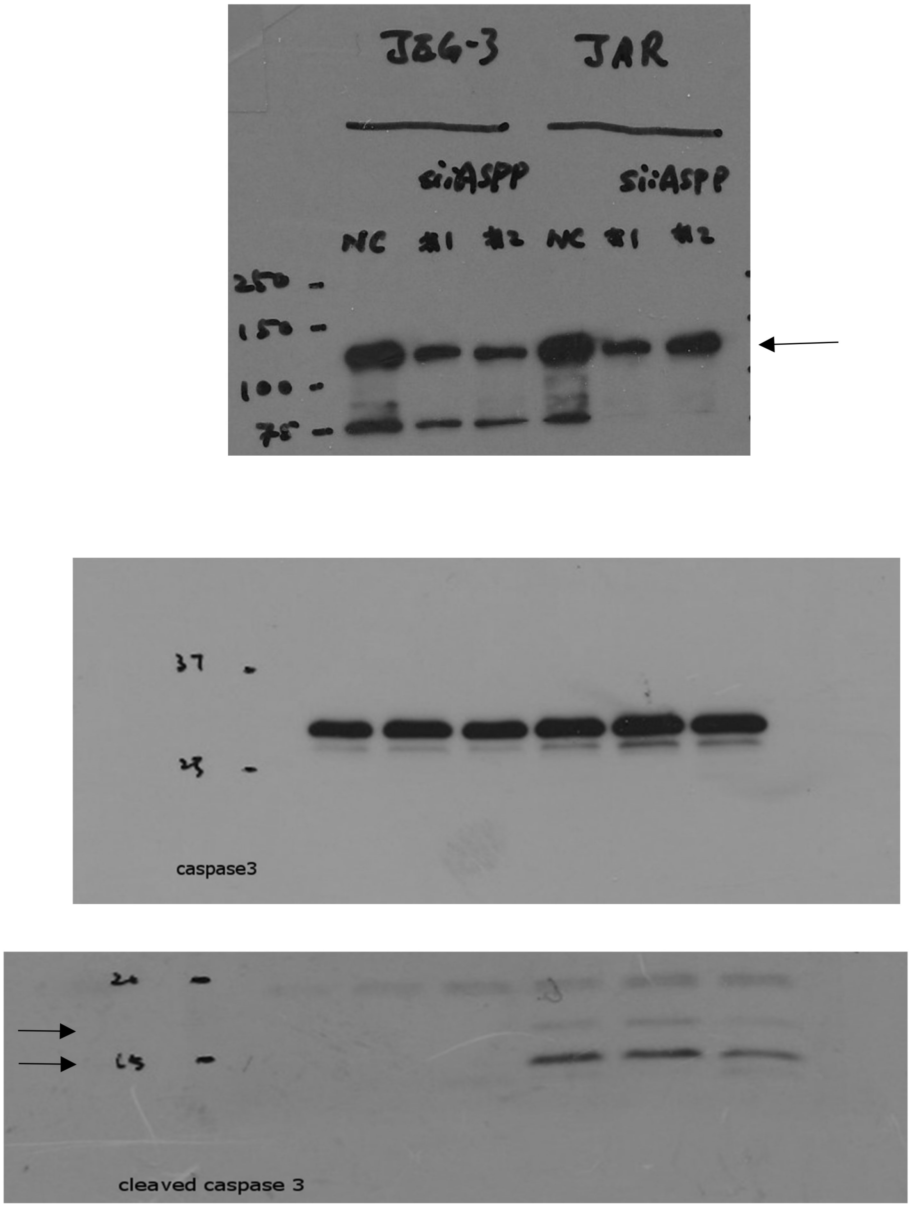
JEG-3
JAR
si:ASPP
si:ASPP
NC #1 #2 NC #1 #2
250
150
100
75
37
caspase3
20
15
cleaved caspase 3

Figure 2D

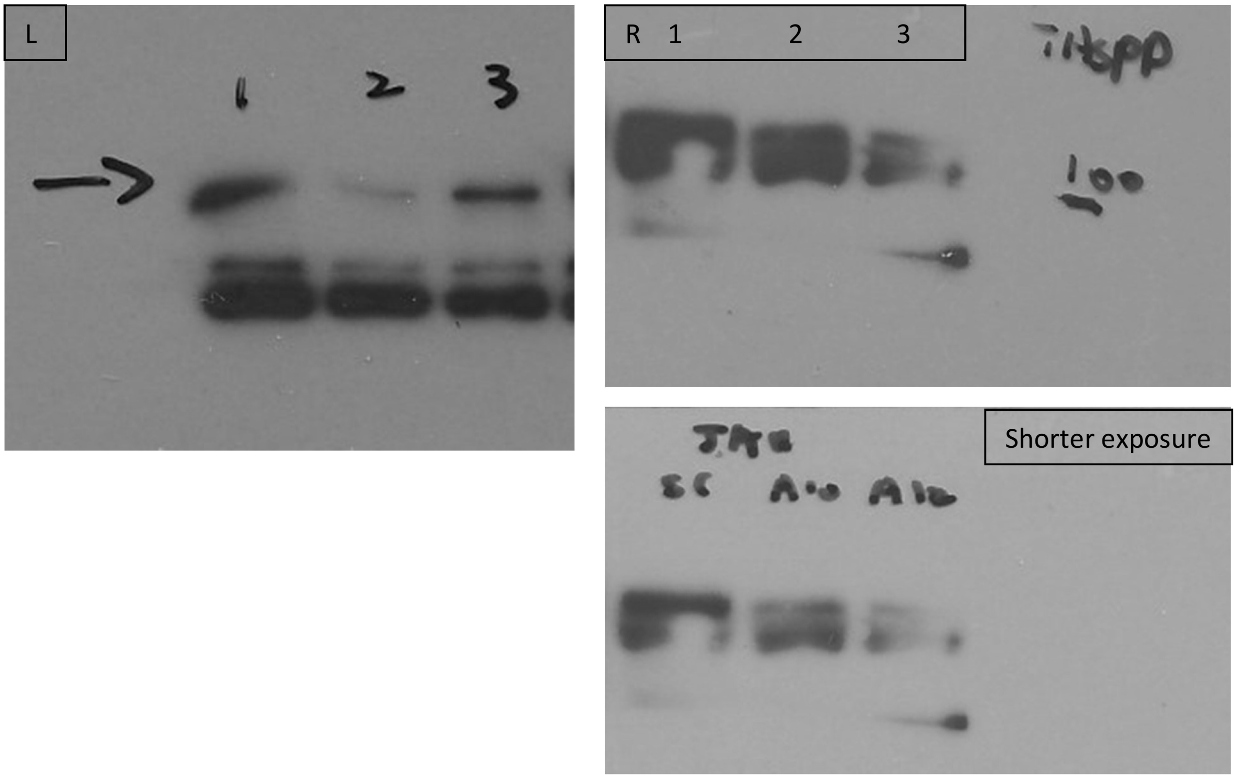

Figure 3D. (L) JEG-3 cells. (R) JAR cells. 1-si, 2-siiaspp#1, 3-siiaspp#2.

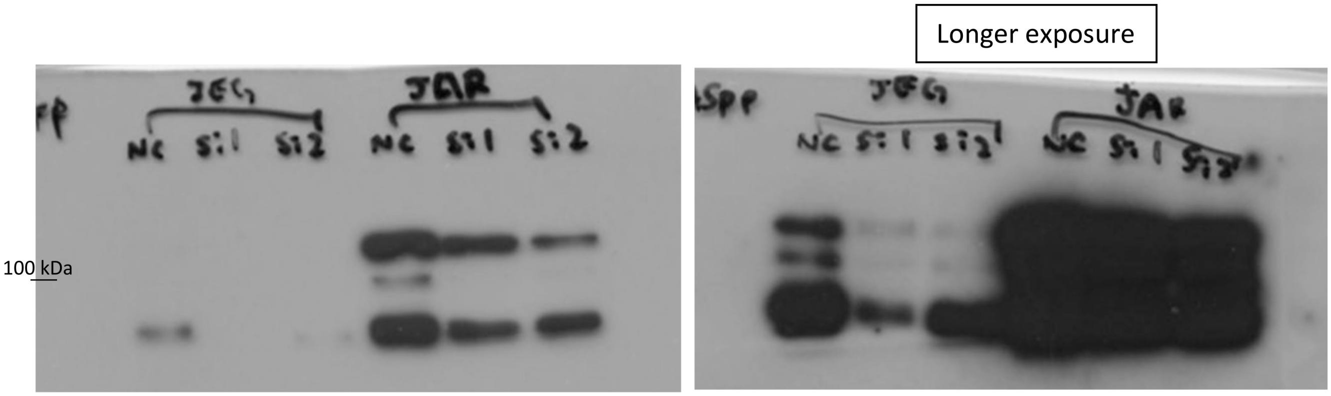

Figure 4C
